# Comprehensive review and assessment of machine learning approaches for host-pathogen protein-protein interaction prediction

**DOI:** 10.1093/bib/bbag051

**Published:** 2026-02-10

**Authors:** Fatima Noor, Muhammad Tahir ul Qamar

**Affiliations:** Institute of Molecular Biology and Biotechnology (IMBB), The University of Lahore, Lahore 54792, Punjab, Pakistan; Department of Bioinformatics and Biotechnology, Government College University Faisalabad (GCUF), Faisalabad 38000, Punjab, Pakistan

**Keywords:** host-pathogen interactions, data integration, machine learning, transfer learning, hybrid models, infectious disease modelling

## Abstract

Predicting host-pathogen protein-protein interactions (PPIs) is a cornerstone of modern infectious disease research, offering unparalleled insights into the molecular mechanisms underlying infection and immune evasion. Despite its transformative potential, the field faces persistent challenges, including limited experimental data, class imbalance, and the dynamic evolution of pathogens. The current study explores cutting-edge computational approaches that have redefined host-pathogen protein-protein interaction (HP-PPI) prediction. Notably, transfer learning has emerged as a game changer, enabling models to leverage knowledge from well-characterized systems to predict interactions in previously underexplored pathogens. Hybrid and ensemble models have proven highly effective, combining the strengths of diverse algorithms to capture the complexity of biological interactions. Explainable AI tools are now bridging the gap between computational predictions and biological interpretability, offering actionable insights into key interaction drivers. Additionally, the review discusses advanced data integration techniques, such as multi-omics fusion and graph-based learning, which explore new dimensions in HP-PPI research. This synthesis of challenges, solutions, and future perspectives highlights a paradigm shift in computational biology, in which scalable, interpretable, and biologically informed models pave the way for breakthroughs in therapeutic discovery, vaccine development, and precision medicine. Our review sets the stage for future advancements, emphasizing the potential of next-generation technologies to unravel the intricate dance between hosts and pathogens.

## Introduction

Host-pathogen protein-protein interactions play a pivotal role in the life cycle of pathogens and the host’s immune response, profoundly influencing infection outcomes [1]. For example, *Yersinia pseudotuberculosis* infects host cells with effector proteins, including YopE, YopT, and YpkA, that degrade the host cytoskeleton and promote bacterial invasion and persistence [2]. In the same way that SARS-CoV-2 uses its spike protein to bind to the host ACE2 receptor, which is essential for cell entry, therefore represents a significant therapeutic target [3, 4]. During infection, the pathogens are capable of avoiding the host’s immune response mechanisms modulating immune signalling, a process that can be facilitated by PPIs in most cases [5]. For instance, bacterial effectors that target host cytokine signalling inhibit the host’s inflammation response [6]. Some viral proteins, such as HIV-1 Nef, disrupt host MHC-I trafficking pathways, thereby preventing antigen presentation and enabling infected cells to evade cytotoxic T-cell-mediated recognition and destruction [7]. These strategies substantiate the notion that host-pathogen PPIs are dynamic and constantly evolving in the face of immune evasion. In response, the host utilizes other PPI networks, which detect, neutralize, and remove pathogens, to combat the pathogen [8]. For instance, Toll-like receptors (TLRs) on host cells bind to pathogen-associated molecular patterns and activate signalling pathways that translate into an inflammatory response and elimination of pathogens [9]. It determines the evolution of the infectious diseases as well as fate of the host in terms of recovery or acquisition of immunity. Indeed, the identification of host-pathogen PPIs is significant for vaccine development [10]. Focusing on critical pathogen-host interactions enables researchers to design vaccines that target these specific points or immune access points [11]. For example, elucidating the SARS-CoV-2-ACE2 interaction facilitated the rapid creation of spike-targeting mRNA vaccines [12]. In a similar manner, research on delivery mechanisms from skin microbiota, including needle-free vaccine formulations, exemplifies the direct application of PPI insights into new immunization methods [13].

PPIs provide multiple promising options for intervention in the development of novel antimicrobials [14]. This network mapping facilitates the identification of central nodes that, when targeted, lead to pathogen elimination. For example, they may interfere with replication or immune response evasion. For example, druggable targets in coronavirus-host interactions have been found through computational modelling for drug development testing [15]. Research on bacterial effector-host interactions has provided a foundation for designing small-molecule inhibitors that disrupt effector function, thereby attenuating virulence [16]. Therefore, understanding host-pathogen interactions through PPIs offers opportunities to advance infection treatment approaches. This is possible due to the combination of computational and experimental techniques that have furthered this evolving domain. Artificial intelligence (AI), and particularly machine learning (ML), is vital due to the intricate nature of host-pathogen networks. ML enables automated evaluation of extensive datasets and integrates various biological information, such as sequences, structures, or expression profiles, turning this information into a self-predicting interaction mechanism. Techniques rooted in the experiments, such as yeast two-hybrid (Y2H) and co-immunoprecipitation (co-IP), lack scalability and sensitivity, but ML does not suffer from these limitations as it can operate across systems and species. Advanced predictive capability for PPI recognition has been achieved using models such as support vector machine (SVM), convolutional neural network (CNN), and graph neural network (GNN), bolstered by cross-species interaction studies, including those involving pathogens.

Host-pathogen interactions occur between organisms from different biological kingdoms in nature, and various machine learning frameworks are being designed to generalize across these systems. Due to this cross-kingdom relevance, this review considers host-pathogen protein-protein interaction (HP-PPIs) involving human, animal, and plant hosts, and pathogens including viruses, bacteria, fungi, oomycetes, and eukaryotic parasites. Plant host-pathogen PPIs, such as those governing effector-target recognition in crop pathogens, share many computational challenges with human HP-PPIs, including a limited number of labelled interactions, class imbalance, species-specific features, etc, making them promising comparative systems. Further, the current study aims to highlight methodological principles across these host types while, where necessary, recognizing system-specific biological considerations by synthesizing approaches from these different host types. Thus, this cross-kingdom perspective allows for a more comprehensive understanding of how current machine-learning methods address the diversity, complexity, and generalizability of HP-PPI prediction.

## Experimental techniques for PPI and their challenges

**Figure 1:**
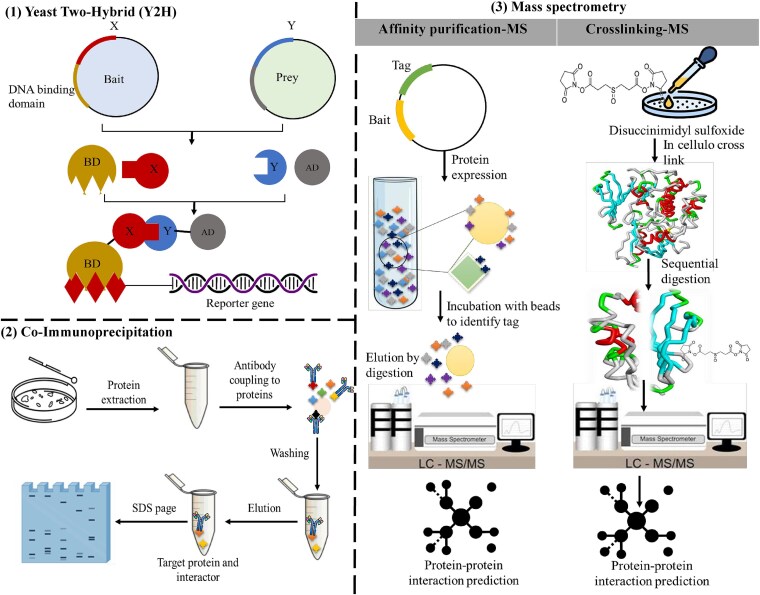
Experimental techniques for protein-protein interaction prediction.

PPIs are crucial to understanding the molecular basis of host-pathogen interactions. The three most common experimental methods used are yeast two-hybrid (Y2H), co-immunoprecipitation (co-IP), and mass spectrometry (MS) (Fig. 1). These techniques face challenges related to throughput, scalability, and the detection of transient or cross-species interactions, which sometimes limit their applicability in complex biological systems.

### Yeast two-hybrid

Yeast two-hybrid is a biochemical assay commonly used in large-scale studies to screen for physical or functional interactions between genes or proteins [17]. This mechanism involves the formation of a functional transcription factor when two proteins combine in the yeast nuclear compartment [18]. However, Y2H has several disadvantages worth noting. The method enables the construction of large-scale interaction screens, which are likely to yield high false-positive rates that require additional validation. These false positives are commonly observed as a result of non-specific and direct interactions arising from high levels of overexpression of the bait and/or prey in yeast [19]. Also, since expressed proteins are not purified, Y2H may yield false negatives, such as proteins that do not get to the yeast nucleus or proteins that do not fold properly in the yeast context [20]. The need to confirm each possible interaction significantly slows its throughput. Y2H is particularly suited to identify meaningful interactions that are sustained for the time required for the reporter gene expression. Transient interactions, which play an important role in processes such as signalling pathways or immune reactions, can hardly be revealed [19]. For example, interactions between signalling adapters or kinases can occur within milliseconds and are not effectively detected by Y2H. While proteins in higher eukaryotes are synthesized with an additional set of amino acids, these proteins may require additional modifications, such as phosphorylation or glycosylation, to attain their functional conformation. This is usually because the yeast system lacks many modifications of these interactions; hence, cross-species interactions are often not noticed. In addition, the basal media used in the yeast laboratory can create a non-physiological environment by cutting out some components of the cellular environment and thus impacting the relevance of the results.

### Co-immunoprecipitation

Co-immunoprecipitation, or Co-IP, is a robust biochemical technique that isolates protein complexes from cell lysates using specific antibodies, thereby identifying interacting partners [21]. However, its utility for large-scale PPI studies is limited by its inherently low throughput [22]. Each co-IP experiment usually requires optimization for the specific protein being studied [23]. In addition, transitory interactions are difficult to detect, as such interactions may dissociate during cell lysis or washing steps and may be excluded from the analysis. Cross-linkers may stabilize such interactions but also introduce non-specific artefacts that complicate interpretation. Studying cross-species PPIs is further hampered by a reliance on high-quality antibodies and compatible expression systems that may not be readily available for non-model organisms or pathogen-specific proteins.

### Mass spectrometry

Mass spectrometry, especially when combined with affinity purification (AP-MS), has emerged as a crucial methodology for large-scale PPI mapping [23]. MS enables the selective identification of proteins in a mixture by using the mass-to-charge ratios of peptide fragments. Yet, this approach poses its own challenges [24]. The throughput and scalability of MS are limited by the need for advanced instrumentation, skilled technicians, and lengthy data analysis processes. Furthermore, sample preparation steps, such as affinity purification and elution, can result in the loss of transient interactions, which are often critical to host-pathogen dynamics [25]. Cross-linking agents can aid in the capture of these transient interactions but also introduce false positives by stabilizing non-physiological complexes. Further, identification of cross-species PPIs using MS is hindered by the need for suitable expression systems and cross-linking strategies, especially with pathogen-specific proteins that do not typically interact with host proteins in experimental contexts. Some other techniques currently used are also mentioned in Supplementary File 1: Table S1. Thus, while Y2H, co-IP, and MS are necessary tools for exploring PPIs, each technique has its drawbacks. Y2H suffers from high false-positive rates and the inability to detect transient, or cross-species, interactions; co-IP is limited by low throughput and the inability to stabilize the dynamic, unfolding complex interaction; MS is resource intensive and requires dedicated protocols to effectively capture complex interaction networks. Combating these challenges often requires a multifaceted approach, combining multiple techniques and computational methods to comprehensively understand host-pathogen interactions.

### Host-pathogen-specific experimental challenges across biological kingdoms

In addition to general factors such as PPI detection, experimental mapping of host-pathogen PPIs is strongly influenced by the biology of the specific host-pathogen system. The secretion systems, post-translational modifications, stage of infection, and biosecurity constraints differ significantly in human and plant hosts and viral, bacterial, fungal, oomycete, and parasitic pathogens. For instance, Xhou *et al*. [26] showed that AP-MS-based maps of SARS-CoV-2–human PPIs require infected mammalian cells under biosafety level 3 conditions. In addition, many bacterial and parasitic effectors only fold correctly or are expressed during infection in their native host context. The limitations present in the two kingdoms, as well as specificity towards the pathogen, help to explain why the datasets are sparse. Therefore, Table 1 summarizes these differences and presents an overview of experimental challenges in HP-PPI mapping, kingdom-wise.

**Table 1:** Comparative summary of biological characteristics and method-specific limitations in host-pathogen protein–protein interaction mapping

Host-pathogen system	Typical HP-PPI features	Limitations of standard Y2H	Limitations of standard co-IP	Limitations of AP-MS/MS-based mapping	Key HP-PPI-specific challenges
**Human-virus**	Kumar *et al*. [27] and Li *et al*. [28] reported that viral proteins hijack host receptors and signalling networks; strong dependence on host PTMs; frequent immune-evasion interactions	Stellberger *et al*. [29] reported that viral proteins often misfold or aggregate in yeast; many require host-specific PTMs (phosphorylation, glycosylation) absent in yeast; membrane or secreted viral proteins are difficult to express	Zhao and colleagues [30] explored that transient or low-affinity immune-evasion interactions dissociate during lysis/washing; overexpression can form non-physiological complexes	Cakir and Li *et al*. [31, 32] mentioned that it requires infected mammalian cells; viral protein toxicity affects viability; difficult to distinguish direct versus indirect interactions	Short-lived and stage-specific interactions; strong cell-type dependence; PPIs involving membrane receptors or viral replication complexes are hard to capture
**Human-bacteria**	Rolando *et al*. [33] reported that secreted/injected effectors target cytoskeleton, immune signalling, transcriptional machinery	Effectors require bacterial chaperones/secretion signals not reproduced in yeast; host and bacterial proteins mislocalize [34]	Bacterial effectors expressed at low levels; weak or transient PPIs lost during lysis; limited antibody availability for pathogen proteins [23]	Requires infection/co-culture; bacterial viability and host cell death complicate preparation; host-bacteria protein mixing complicates MS [35]	Cross-kingdom folding/PTM mismatch; effector expression restricted to infection stage; difficulty separating direct effector-host PPIs from complex assemblies
**Human-parasite**	Multi-stage life cycles; organelle-targeted proteins; exported effectors modulate immunity [36]	Stage-specific or organelle-targeted proteins mislocalized in yeast; large low-complexity regions impair folding according to Cuesta-Astroz *et al*. [37]	Parasite proteins unstable outside native environment; co-IP from mixed host-parasite lysates suffers from low parasite:host ratios [36]	Parasites require specialized culture; low biomass reduces MS sensitivity; enrichment of parasite proteins is technically difficult [36]	Strong stage specificity; tissue tropism; extreme host:parasite protein imbalance; challenging enrichment without losing host context
**Plant-fungus**	Secreted apoplastic/nuclear effectors; targeting of plant immune receptors, TFs, signalling hubs [38]	Plant receptors and fungal effectors misfold or mislocalize in yeast; apoplastic/chloroplast interactions not recapitulated [39, 40]	Plant immune complexes are transient and triggered by recognition; extraction disrupts receptor-effector complexes [41]	Infection must be performed in planta; separating host versus fungal proteins is difficult; strong stage-specific expression [42]	Compartment-specific (apoplast, chloroplast, nucleus) PPIs; dependence on infection stage and plant genotype; challenging redox/ion homeostasis *ex vivo*
**Plant-oomycete**	RxLR/CRN effectors target immunity hubs; strong spatial/temporal regulation; secretion only during infection [42]	RxLR processing, secretion, and host entry signals absent in yeast; effectors fail to reach correct compartment [43]	Effector-receptor PPIs transient and stage-specific; low expression in heterologous systems [44]	Oomycete growth and effector expression often limited to infected tissues; dual-organism samples complicate MS [45]	PTM and secretion differences; strict in planta expression; dependence on immune status; difficulty replicating natural infection environment
**Plant-bacteria**	Type III effectors, TAL effectors, toxins; many target transcription and immunity [46, 47]	TAL and repeat-rich effectors misfold; plant-specific cofactors missing; nuclear targeting not reproduced in yeast [48]	TAL-DNA and TF interactions lost after lysis; weak interactions hard to stabilize [49, 50]	Requires infected tissues; nuclear fractionation adds complexity; low-abundance regulators difficult to detect by MS [51]	Nuclear and plasma-membrane PPIs; dependence on plant development/environment; intact host-pathogen interaction required

## Machine learning foundations for PPI prediction

ML has transformed the way we predict PPIs, providing powerful tools for analysing extensive biological datasets and revealing patterns that might be difficult to detect using experimental techniques alone. The typical workflow for ML-based PPI studies includes several key steps: dataset preparation, feature extraction, model selection, training, and validation. Each of these steps plays a vital role in ensuring the accuracy and interpretability of the predictions.

### Dataset preparation

The foundation of ML models lies in high-quality datasets. Experimental databases such as BioGRID, STRING, and IntAct [52–54] provide curated PPI information across various organisms. However, the raw data from these sources often needs preprocessing to guarantee the reliability of the model. A major challenge is the class imbalance present in PPI datasets, where the number of interacting protein pairs greatly exceeds that of non-interacting pairs [55]. This imbalance can skew ML models, necessitating the creation of synthetic negative samples. For example, Yu *et al*. [55] developed a negative dataset by pairing proteins that were unlikely to interact. Furthermore, noise in the data stemming from low-confidence interactions or experimental errors requires preprocessing steps such as deduplication and the elimination of false positives to enhance model performance.

### Feature selection

**Figure 2:**
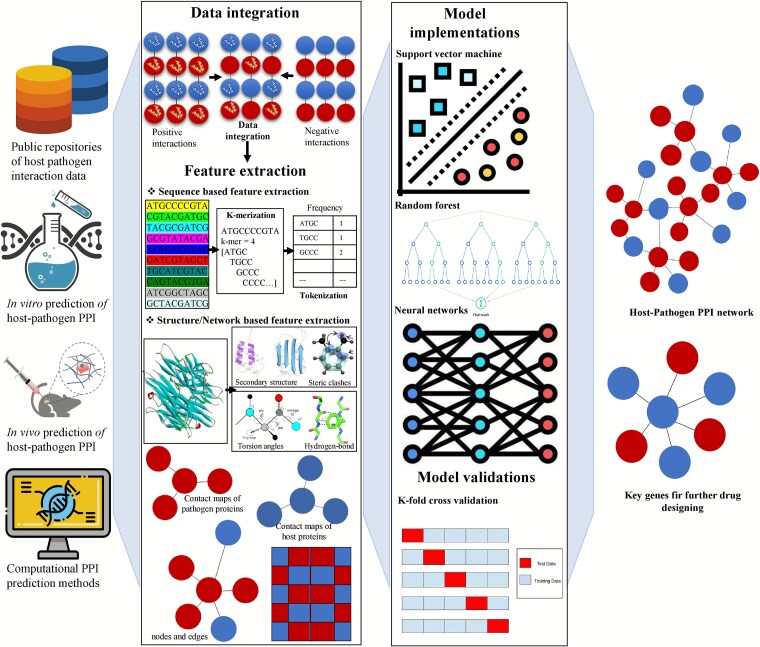
Overview of the HP-PPI prediction workflow combining experimental data, feature extraction (sequence, structure, function, networks), and computational models (e.g. ML, network-based). Host and pathogen proteins are represented as distinct node types, with predicted interactions shown as edges, illustrating integrated strategies for accurate interaction prediction.

Feature selection is one of the most crucial steps in the ML for predicting PPIs [56, 57]. It refers to the process of converting received protein data into a set of numerical features usable by machine learning algorithms. By identifying basic biological and structural features, feature extraction helps models distinguish between interacting and non-interacting protein pairs [58]. Sequence-based features are mainly derived from protein sequences, while structure-based features stem from the tertiary structures of proteins, and function-based features are associated with protein function in protein interactions. Among the common features, sequence-based features [59] are particularly popular because they are relatively easy to compute (Fig. 2). Some of these features include the amino acid content, which measures the percentage composition of each amino acid in a protein sequence; and the dipeptide or tripeptide content, which considers patterns of consecutive two or three amino acids that are important in interface regions of interacting proteins. For instance, a primary structure may incorporate specific motifs such as Gly-Pro-His that would show that it binds with specificity. Furthermore, parameters such as hydrophobicity, charge, and molecular weight reveal biophysical properties of protein sequences. The predictions are refined using position-specific scoring matrices (PSSMs) derived from sequences and alignments, which incorporate additional information about sequence conservation, a property commonly associated with functional regions. Sophisticated methods like k-mer encoding split sequences into overlapping chunks and detect motifs that point to interaction propensity.

Structure-based features [60] rely on the three-dimensional conformation of proteins to offer spatial and biophysical props. Some of these features are docking scores that help in measuring the binding energies between proteins by employing methods such as HADDOCK [61] or AutoDock [62], and spatial relationships that include solvent accessible surface area and inter-residue distance. Another important facet for structural predictions is interaction hot spots, individual amino acid residues or clusters on the surface of the protein that are substantially involved in the binding process. Further, percentages of the secondary structures like helices, sheets, and coils are informative as these structural components participate in the formation of interaction sites. Another important type of features is dynamic, which include residue flexibility and conformational changes, obtained from molecular dynamics simulations, and are beneficial to capture transient interactions.

Function-based features [63] provide additional high-level annotations of protein roles and the molecular pathways in which these proteins are involved. Common tools for achieving this include the Gene Ontology (GO) terms, which provide information on molecular functions, biological processes, and cellular components of proteins. It is crucial that interacting proteins share at least one GO term, as this suggests they are involved in similar pathways. Likewise, pathway annotations from the KEGG [64] or Reactome [65] databases add functional context by pointing to proteins involved in the same signalling or metabolic pathways. Information on domains and motifs like the existence of SH2 domains or phosphate sites also gives a signal of the likelihood of interaction. Other important characteristics include subcellular localization since proteins in the same compartment are very likely to interact.

The fusion of sequence, structure, and function-based features has been found to yield high accuracy for PPI prediction (Supplementary File 1: Table S2). For example, it is possible to enhance transient interaction detection by including sequence embeddings alongside structural information or to give contextual information by integrating functional annotations. However, feature extraction is not without challenges, as discussed in detail in the following section. For instance, structural data may be scarce for many proteins, restraining the use of structure-based features. Similar to phenotypic annotations, functional annotations may also be erroneous or contain missing data, especially for non-model organisms. Also, obtaining structure-based features like docking scores or molecular dynamics simulations can be computationally expensive for high-dimensional datasets.

### Model selection

Choosing a suitable ML model is the initial and most critical decision in PPI prediction, as it defines the approach to analysing protein properties and modelling interactions. Previous works using the binary classification framework for PPI studies have employed features learned by ML models such as SVMs and Random Forests. The SVMs are more suited for the high dimensions of data and compatible with the features such as amino acid composition, physicochemical properties, and evolutionary conservation. Random Forests, on the other hand, can provide robust solutions for noisy or imbalanced data, with interpretable predictions via feature importance rankings. Though the traditional models are computationally efficient and easy to interpret, these suffer from shortcomings, especially when the patterns are complex and non-linear, such as in PPI data. In order to overcome these challenges, deep learning models are playing an influential role in the present days. CNNs are good at mapping spatial features over linear protein sequences or even their 3D structures, whereas GNNs learn proteins as nodes and their interactions as edges, considering both local and global features. Long short-term memory (LSTM) networks, which are a subcategory of recurrent neural networks (RNNs), are well suited for processing sequential data such as protein domains and motifs. Approaches that combine traditional and deep learning techniques have also been developed as possible methods that leverage both.

### Model training

The process of training, after choosing a model, entails exposing the model to certain patterns associated with protein interactions using labelled datasets [66]. This is done by splitting the datasets into training, validation, and testing sets, which are traditionally carried out in a stratified fashion to ensure that the interacting and non-interacting pairs are in equal proportion. During training, optimization algorithms are used to drive the parameters of the model closer to their optimal values using stochastic gradient descent or Adam optimizers, and a loss function like binary cross-entropy to measure the error in prediction. The model learns by minimizing the loss function through iterative parameter updates. It updates parameters iteratively using the gradient of the loss function:


$$ {\theta}_{t+1}={\theta}_t-\eta{\nabla}_{\theta }L\left(\theta \right) $$

where ${\nabla}_{\theta }L\left(\theta \right)$ is the gradient of the loss function, $\eta$ indicates learning rate and experiences slow convergence when dealing with complex biological datasets that contain noisy features, such as sequence embeddings or structural annotations. To tackle these challenges, researchers have increasingly turned to adaptive optimizers like Adam. The Adam optimizer updates the learning rate for each parameter using first- and second-moment estimates of the gradients.


$$ {m}_t={\beta}_1{m}_{t-1}+\left(1-{\beta}_1\right){\nabla}_{\theta }L\left(\theta \right) $$

$$ {v}_t={\beta}_2{v}_{t-1}+\left(1-{\beta}_2\right){\left({\nabla}_{\theta }L\left(\theta \right)\right)}^2 $$

$$ {\hat{m}}_t=\frac{m_t}{1-{\beta}_1^t} $$

$$ {\hat{v}}_t=\frac{v_t}{1-{\beta}_2^t} $$

$$ {\theta}_{t+1}={\theta}_t-\frac{\eta }{\sqrt{{\hat{v}}_t}+\in }{\hat{m}}_t $$

Given Adam’s adaptive nature, this may be especially applicable to host-pathogen studies in which feature importance might differ drastically across species’ proteins. Some studies such as Fout *et al*. [67] had successfully implemented the use of Adam-trained GCNs for protein interface prediction, thus resulting in performance improvement over classical gradient descent techniques. Further, the choice of a loss function is crucial in guiding the optimization process. Binary cross-entropy (BCE) is the most common loss function used in PPI prediction tasks, where the goal is to classify protein pairs as interacting ($y=1$) or non-interacting ($y=0$). BCE quantifies the error between predicted probabilities ($\hat{y}$) and actual labels ($y$):


$$ {L}_{\mathrm{BCE}}=-\frac{1}{N}\sum_{i=1}^N\Big[{y}_i\log \left({\hat{y}}_i\right)+\left(1-{y}_i\right)\log \left(1-\hat{yi}\right) $$

In cases of imbalanced datasets, weighted BCE is used to assign higher penalties to misclassified positive samples (interactions). Weights are calculated using ${w}_1$ and ${w}_0$


$$ {L}_{\mathrm{weighted}}=-\frac{1}{N}\sum_{i=1}^N\Big[{w}_1{y}_i\log \left({\hat{y}}_i\right)+{w}_0\left(1-{y}_i\right)\log \left(1-\hat{yi}\right) $$

One of the major challenges in ML models is overfitting, especially in PPI studies with high-dimensional data, such as protein sequences or structural embeddings. Regularization techniques reduce overfitting by constraining the model’s complexity.


$$ {L}_{\mathrm{reg}}=L+\frac{\lambda }{2}\sum_{j=1}^P{\theta}_j^2 $$

The model parameters are indicated by ${\theta}_j^2$, while $\lambda$ is the regularization strength. Singh *et al*. effectively employed regularization in their model for the prediction of drug targets in microbial-associated cardiovascular diseases by incorporating host-pathogen interaction network parameters. Various strategies, such as oversampling the minority class, undersampling the majority class, and generating synthetic interactions via algorithms like SMOTE, are employed to address this issue. Another form of weighted loss function is used, assigning higher penalty values to misclassified interacting pairs to improve model sensitivity. Further, data augmentation techniques like introducing noise or perturbing the protein features are applied to simulate diverse biological scenarios and improve model robustness.

### Model validation

Model validation ensures the accuracy, reliability, and generalizability of predictions. One popular technique is cross-validation, where a dataset is split into multiple folds; in each iteration, the model is trained on all but one fold, which is used for validation. In $k$-fold cross-validation, the dataset is split into $k$ equally sized subsets (folds). In each iteration, the model is trained on $k-1$folds and validated on the remaining fold. The process repeats $k$ times, and the final performance metric is averaged:


$$ \mathrm{CV}\ \mathrm{score}=\frac{1}{k}\sum_{i=1}^k{M}_i $$

Validation is critical for HP-PPI prediction to maintain model reliability and prevent overfitting. Common evaluation metrics include accuracy, precision, recall, F1 score (harmonic mean of precision and recall), AUC-ROC (Area Under the Receiver Operating Characteristic curve), and AUC-PR (Area Under the Precision-Recall curve). In evaluating F1 score, it is often considered the most effective for imbalanced datasets because it averages precision and recall, whereas AUC-PR is also focused on the positive class, which occurs in rare interactions. Model performance can also be improved through hyperparameter tuning, including the learning rate and model structure, as well as grid, random, or Bayesian optimization. Validation has proven essential in various studies. Fout *et al*. [35] provided evidence that GCNs outperform traditional approaches by capturing complex dependencies within interaction networks. Xu and Wojtczak [68] illustrated how functional annotations, together with multi-channel neural networks, were integrated to reveal immune-evasion strategies in interactions of the influenza virus with its host. Gordon *et al*. [69] prioritized SARS-CoV-2-host interactions using AP-MS data and machine learning, including Random Forests, to identify potential drug targets such as ORF8-HDAC2. Yang *et al*. [70] proposed the use of deep learning to combine evolutionary profiles and a fully trainable Siamese CNN and MLP, incorporating transfer learning to enhance generalization. Shakibania *et al*. [71] utilized deep learning alongside the monoMonoKGap feature extraction technique to achieve high accuracy across human-pathogen datasets. Yang *et al*. [72] noted the application of deep learning to prospective human-virus PPI prediction, emphasizing its relevance for understanding the mechanisms of viral infections and enabling drug development.

### Practical workflow for constructing HP-PPI predictors

Predicting host and pathogen protein-protein interaction requires a methodology that extends beyond classical intra-species PPI modelling. As interacting partners arise from different evolutionary lineages, differ in sequence composition, and often function in compartmentalized host environments, HP-PPI prediction requires customized procedures for dataset construction, feature engineering, model training, and evaluation. This study for the first time describes a harmonized and evidence-based workflow to develop robust HP-PPI predictors across different host-pathogen systems.

#### Dataset curation and integration

To construct HP-PPI predictors, the first step is carefully curating verified interactions from the literature. Information on HP-PPI is thin and scarce for many pathogens; therefore, interactions from different sources should be integrated. Public databases such as HPIDB, VirHostNet, PHISTO, and BioGRID contain curated cross-kingdom interactions. They come from yeast two-hybrid screens, affinity purification–mass spectrometry assays, and small-scale targeted experiments. Ammari *et al*. [73] and Guirimand *et al*. [74] have pointed out that these repositories differ in depth of annotation, confidence in experiments, and host-pathogen coverage. To ensure a consistent way of representing these interactions across the host and pathogen taxonomic groups, harmonization steps such as removal of redundant isoforms, unification of protein identifiers, and evidence code-based filtering are required. Several studies, including Alam *et al*. [75] and Volzhenin *et al*. [76], show that the accuracy of the prediction is highly influenced by dataset quality, especially for models based on deep representation learning.

#### Negative sampling method for cross-kingdom interaction

The definition of a negative sample is much more challenging for HP-PPIs than for intra-species PPI prediction, as true non-interacting pairs are seldom confirmed experimentally. Getting a random sample is a naive process that creates biologically implausible negatives. This also introduces a strong bias to the dataset. Recent studies utilizing computational approaches, such as Li *et al*. [77], aim to reduce this tendency through more biologically relevant means. For example, checking for subcellular localization incompatibility (e.g. secreted pathogen effector versus cytosolic host enzyme), functional incongruence filtering, taxonomic separation, and known orthologous interaction pairs exclusion. These methods limit the chances of labelling unknown positives as negatives. More advanced approaches use prior biological information regarding either gene expression non-coherence, evolutionary divergence, or structural incompatibility, as Li *et al*. [77], did in their HP-PPI deep learning framework.

#### Cross-species feature harmonization

The representation of features is a very important aspect of making the models generalize across species. Many handcrafted descriptors, such as amino acid compositions, pseudo amino acid features, and autocorrelation index, fail to accurately reflect the structural diversity of pathogen effectors or the higher-order functional constraints in host proteins. New HP-PPI techniques employ representation learning using a protein language model or structural embeddings. According to research by Rives *et al*. [78] on ESM models, Elnaggar *et al*. [79] on ProtTrans, and Lin *et al*. [80], transformer-based embeddings capture important biological and chemical signals that can travel across kingdoms. When used with predicted structure from AlphaFold2 or evolutionary couplings [81, 82], such embeddings greatly improve model performance for HP-PPIs. Aligning feature spaces across species guarantees that the model encodes signals relevant for interaction.

#### Addressing severe class imbalance

The datasets HP and PPI are badly imbalanced as data indicate that positive interactions are <1%-2% of all possible protein pairs. When the data are unbalanced, they can severely affect model learning, leading to trivial classifiers that are overly influenced by the majority class. To overcome this, researchers have adopted several strategies, including weighted cross-entropy, focal loss, and controlled undersampling of non-interacting pairs. Using class weighting in studies like Du *et al*. [83] effectively boosted the capacity of deep models to identify pathogen-host interactions. We can augment the minority class without altering the underlying feature distributions using advanced sampling techniques such as biologically constrained SMOTE or density-aware oversampling. These methods reduce overfitting and promote robust representation of true positive interactions.

#### Protocols and tests for biological generalizability training

Randomized train-test splits are inadequate for HP-PPIs, as they artificially inflate performance by including homologous pathogen proteins in both sets, contrary to intra-species PPI prediction. HP-PPI studies now utilize cross-validation strategies that leave one pathogen out for the family. For example, LOPO (leave one-pathogen protein out) evaluation better predicts a model’s ability for the novel pathogen, Abbasi *et al*.’s [84] findings. Chen and coworkers also propose cross-kingdom validation: training an RNN-based approach on human-virus interactions and testing it on plant-fungus data to assess the model’s robustness across broader biological interactions. The complex assessment frameworks reflect realistic use cases like predicting interactions for new viruses or newly sequenced plant pathogens.

#### Interpretability and biological validation

The use of HP-PPI models for translation requires interpretability. Deep learning methods are integrating interpretability tools, such as SHAP, integrated gradients, or attention-weighted visualizations, into their own sample outputs to revert predictions back to useful, biologically meaningful residues or motifs. Research conducted by Lundberg *et al*. [85] and Ling *et al*. [86] demonstrates that interpretable models can discover structural hotspots, immune-evasion motifs, and functional interfaces in the proteins of hosts or pathogens. According to the common scenario, motif enrichment analysis, comparison with known host signalling pathways, and docking studies to verify spatial plausibility are part of the downstream biological validation. The combination of these steps leads to mechanistic insights from machine learning.

## Traditional ML techniques

### Support vector machine for predicting host-pathogen PPI

SVM is a traditional technique of machine learning that is often used for classification problems with a binary outcome variable, such as whether a given protein pair interacts or not [52]. SVMs can be effective even in high-dimensional feature spaces and perform well on datasets where relationships between input features are complex [53]. The goal of an SVM is to find an optimal hyperplane that maximizes the margin of separation between points belonging to two different classes [54]. The mathematical formulation of the SVM optimization problem is as follows:

${\displaystyle \begin{array}{c}{minimized}_{w,b}=\dfrac{1}{2}{\left\Vert w\right\Vert}^2\\{}\kern6em \end{array}}$ subject to ${y}_i\left({w}^T{x}_i+b\right)\ge 1,i=1,\dots N$.

$w$ represents the weight vector that defines the orientation of the hyperplane. Bias term that determines the hyperplane’s position is represented as $b$. The ${x}_i$indicates the feature vector, and interaction and non-interaction are represented with +1 and −1, respectively. In practice, datasets often contain overlapping or noisy data points, making it impossible to find a perfect separating hyperplane [87]. To address this, SVMs use a soft-margin approach by introducing slack variables $\left({\varepsilon}_i\right):$

${\displaystyle \begin{array}{@{}c}{minimized}_{w,b,\varepsilon }=\dfrac{1}{2}{\left|\left|w\right|\right|}^2+\mathrm{C}\displaystyle\sum_{i=1}^k{\varepsilon}_i\\{}\kern10em \end{array}}$ subject to ${y}_i\left({w}^T{x}_i+b\right)\ge 1-{\varepsilon}_i, {\varepsilon}_i>0,i=1,\dots N$.

$C$ is a regularization parameter [88] that controls the trade-off between maximizing the margin and minimizing classification error. SVM also handles non-linear relationships between features using kernel functions that map the input data into a higher-dimensional feature space. The SVM-based HP-PPI models differ from conventional PPI applications primarily in the features and sampling strategies required to capture asymmetry between host and pathogen proteins. To train SVM models effectively, it is essential to use cross-kingdom harmonized feature vectors or descriptors. These include, but are not restricted to, conjoint triad descriptors, evolutionary profiles, secretion-signal-aware motifs, and effector-specific biochemical signatures distinguishing pathogen proteins from host regulatory proteins. Numerous studies have shown that low-complexity regions [89], cysteine-rich and translocation peptides of pathogen effectors [90], and the SVM feature representation greatly improve model separability.

Another key adaptation is the use of pathogen-aware kernel functions, which permit distinct non-linear mappings between host sequences and pathogen sequences with different evolutionary rates [71]. Weighted radial basis kernels, also known as domain-specific spectrum kernels, were used to amplify the contribution of conserved interface residues and suppress noise from unassociated regions. HP-PPI datasets are very unbalanced. Therefore, SVMs are usually trained with class-weighted margins, distance-aware penalties, or hard-negative filtering to improve sensitivity to rare but biologically important interaction pairs.

Recently, Cui *et al*. [91] also proposed an approach for identifying viral-host PPIs using an SVM that combined multiple features, including protein sequences and functional domains. Their model offered a high accuracy rate indicating the ability of SVMs in discovering important interactions that lead to viral infections. Basit *et al*. [92] examined large-margin machine learning models particularly SVMs to predict HP-PPIs. These studies showed that SVM performance is highly dependent on the quality of negative sampling. Using protein pairs that are incompatible with localization or functionally different as negatives, rather than random pairs, yields a more biologically realistic decision boundary. The successful implementation of SVMs in HP-PPI prediction is more reliant on HP-specific feature extraction, kernel designing, and sampling scheming, rather than the classifier itself. SVMs have been used to identify host-pathogen PPIs, helping elucidate infection pathways and potential targets for intervention. Wang *et al*. [61] used sequence-based SVM models to predict PPIs *in Plasmodium falciparum* and *Escherichia coli*, achieving accuracies of 93.8% and 95.3%, respectively. Their approach included careful selection of negative samples, highlighting SVMs’ effectiveness in HP-PPI prediction and their potential to guide therapeutic development.

### Random Forests

Random Forests are popular for HP-PPI modelling as they can handle heterogeneous biological features and are robust to noise and missing annotations, which are common in pathogen genomes. HP-PPI apps usually use RFs to aggregate sequence patterns and predict secretion signals, subcellular localization, domain-domain compatibility scores, and functional annotations (e.g. host immune pathway terms and pathogen virulence factors). Various features residing in RFs combine to model hierarchical biological interactions that would not be captured using a single feature type alone. Random Forests work by training a multitude of decision trees and then aggregating their predictions to generate a final output [62, 93]. In binary classification problems, such as HP-PPI prediction, the class is determined by majority voting. Each decision tree predicts a class ${C}_t$ for a given protein pair $x$.


$$ {C}_t(x)={\mathrm{argmax}}_{c\in \left\{0,1\right\}}\ P\ \left(c|x,{\Theta}_t\right) $$

$P$ is the probability of class $c$ given input $x$ and tree parameters ${\Theta}_t$. Feature importance in Random Forests is measured using metrics such as Gini importance or mean decrease in impurity. For feature, its importance is calculated as the average decrease in node impurity across all trees:


$$ Importance\ \left({f}_j\right)=\frac{1}{T}\sum_{t=1}^T\sum_{n\in Nodes(t)}\Delta{Impurity}_n\left({f}_j\right) $$

$\Delta\ {Impurity}_n\left({f}_j\right)$ is the reduction in impurity at node $n$ split by feature ${f}_j$. Li *et al*. [64] proposed an RF-based method that integrates minimum redundancy maximum relevance and incremental feature selection for the prediction of protein-protein interaction sites. This approach achieved a 82% success rate on a benchmark dataset of 180 proteins, thereby highlighting the utility of RF for identifying critical interaction sites. Dyer *et al*. [65] and Saha *et al*. [94] employed RF to predict host-pathogen interactions and potential drug targets in *P. falciparum*, respectively. Even though RF models are generally resistant to noise and fusion of different data types, they tend to struggle with many issues including overfitting, class imbalance, and high computational costs. These issues can be addressed through restricting tree depth, data partitioning for parallel processing, and feature pruning. The addition of biological context, SHAP [95], and explainable AI (XAI) tools [96] improves the interpretative power of the model, strengthening the rationale for using RF in HP-PPI research.

### Decision trees

In HP-PPI prediction, decision trees are primarily used to extract biologically interpretable rules. The design helps the model learn hierarchically structured decision rules such as presence of an RXLR motif, specific combinations of GO terms, and host-pathogen domain complementarity, which map on to known infection mechanisms. Decision trees were particularly useful in HP-PPI prediction pipelines compared to SVMs and RFs because they could predict explicit molecular determinants of binding. Decision trees works by recursively partitioning the data space based on feature values, resulting in a tree-like structure in which each internal node represents a decision rule, and each leaf node denotes an outcome. This hierarchical structure assists in the ease interpretation, and hence, particularly useful in domains where model transparency is crucial [97]. To build a decision tree, at each node, one has to pick the feature that best splits the data. It depends on which impurity measure one is considering like Gini impurity or entropy. For a dataset with classes $C1,C2,C3,\dots CK$


$$ G=1-\sum_{i=1}^K{p}_i^2 $$

${p}_i$ is the proportion of samples belonging to class ${C}_i$ at that node. Decision trees are a good candidate for host-pathogen PPI analysis because they are simple, interpretable, and robust to diverse feature sets [71]. For HP-PPIs, in particular, data from sequence, structural, functional, and evolutionary sources can be integrated into decision trees to uncover patterns and rules that govern host-pathogen protein interactions. It is precisely the hierarchical structure of decision trees that recommends them for the understanding of the underlying biology of these interaction since the rules at each node may reveal key determinants of interaction specificity.

By leveraging cross-kingdom features, decision trees can identify biomedically meaningful splitting criteria (e.g. a pathogen effector is secreted, a host protein is nuclear, and both share kinase-related GO terms) that reflect mechanistic patterns of pathogen manipulation of host signalling pathways. Therefore, while single decision trees may be weaker compared to ensemble methods, they serve as an important tool in designing HP-PPI models that are interpretable as well as generating hypotheses.

## Deep learning approaches in host-pathogen PPI prediction

Deep learning has significantly improved HP-PPI prediction, enabling models to learn representations that reflect evolutionary signals, structural constraints, and interaction-specific biochemical signatures without relying on crafted features. Compared with general PPI tasks, HP-PPI prediction requires the model to handle cross-kingdom divergence, immune-evasion strategies, pathogen effector specialization, and scarce training data. The following subsection highlights how CNNs, RNNs, and GNNs have been adapted for HP-PPI prediction.

### Convolutional neural networks for capturing spatial and sequential patterns

**Figure 3:**
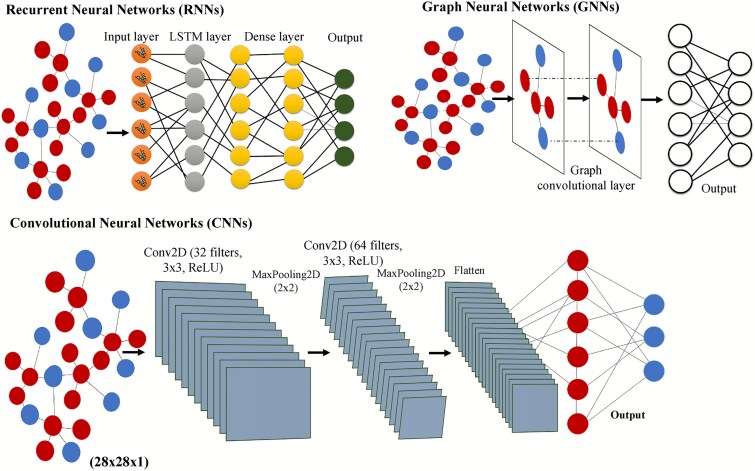
A schematic representation of a multi-deep learning network architecture for host-pathogen protein-protein interaction (HP-PPI) prediction. The inputs are processed through multiple stacked layers of deep learning networks, each layer refining feature representations and learning complex interaction patterns. The final output layer predicts interactions, with distinct node types indicating the predicted connections between pathogen and host proteins. This architecture highlights the use of deep learning to enhance the accuracy of HP-PPI predictions.

Due to their proficiency in handling organized biological data, CNNs are particularly effective for host-pathogen PPI prediction (Fig. 3) [98]. CNNs are capable of performing advanced pattern recognition because protein sequences can be symbolized as 1D vectors via k-mer embeddings, PSSMs, or physicochemical property vectors [99]. With respect to structural data, protein structures provide 2D inputs in the form of contact maps and co-evolution matrices, as well as 3D spatial distance matrices, which enable CNNs to capture complex spatial features [100]. Moreover, CNNs study interaction networks through adjacency matrices in a hierarchical fashion [100]. In the same case of 1D input, protein sequences are represented as numerical vectors, such as k-mer embedding, PSSM [101], or physicochemical property vectors. In the same case of 2D input, a contact map or a co-evolution matrix is used to incorporate structural information. In the same case of 3D input, spatial distance matrices are derived from protein structures to assist CNNs to learn complex geometric features. After input preparation, a convolutional layer applies filters ($W$) to the input ($X$), producing feature maps ($F$):


$$ {F}_{i,j}\sum_{m,n}{W}_{m,n}.{X}_{i+m,j+n}+b $$

${W}_{m,n}$ is the filter weights, ${X}_{i,j}$ is the input data, and $b$ represents the bias term. After convolutional operations, pooling layers (e.g. max pooling or average pooling) reduce the dimensionality of feature maps, improving computational efficiency while retaining important features:


$$ {P}_{i,j}=\max \left\{{F}_{i+,k,j+1}\right|k,l\in \mathrm{pooling}\ \mathrm{window} $$

Later, the extracted features are flattened and passed through fully connected layers, culminating in a final prediction:


$$ \hat{y}=\sigma \left(W.h+b\right) $$

In that equation, $\hat{\ y}$ is the predicted probability of interaction, $h$ indicates the flattened feature vector, and the activation function is represented with $\sigma$. Finally, the model outputs interaction probabilities for protein pairs, enabling binary classification (interaction versus non-interaction). Chen *et al*. [102] introduced an innovative framework, PIPR (Protein-Protein Interaction Prediction using Siamese Residual CNN), which predicts PPIs using only amino acid sequences. PIPR employs a deep residual recurrent CNN within a Siamese architecture, enabling the extraction of local features while retaining the contextual information of sequences without relying on predefined features. Similarly, Zeng *et al*. [103] developed DeepPPISP, a TextCNN model that applies a sliding-window approach to merge global and contextual sequence features. One of the more challenging tasks involves preparing intricate biological information for CNNs, although incorporating AlphaFold-based features significantly enhances the input. The issue of class imbalance present in HP-PPI datasets can be solved using a combination of weighted loss functions and SMOTE. The black-boxness problem of CNN’s interpretability can be solved with Grad-CAM, which gives some visibility to the regions within the sequences or structures guiding the model’s predictions. The continual increase in multi-omics datasets necessitates the use of scalable architectures on powerful GPUs. While these issues exist, CNNs remain fundamental to the HP-PPI prediction problem considering their ability to utilize spatial and temporal relations that are important for infection biology and therapeutic development. CNNs have emerged as one of the most widely used architectures for HP-PPI modelling but have many practical limitations restricting their use in cross-kingdom interaction data. CNNs can often suffer from limited interpretability. This is because the convolutional filters capture complex local patterns that do not easily map back to specific residues, motifs, or other biologically meaningful regions in host or pathogen proteins. CNNs are also very sensitive to extreme class imbalance, which is common in HP-PPI datasets, where the number of true interacting pairs is much smaller than that of non-interacting pairs, leading to biased predictions. The proteins of hosts and pathogens are usually heterogeneous in nature. This presents a challenge as the host proteins tend to be longer, domain rich, and structurally complex. In contrast, the effector and viral proteins of pathogens are often short, disordered, and dominated by linear motifs. This discrepancy complicates uniform input encoding. In conclusion, the performance of CNN may deteriorate by structural inputs like weak contact maps or AlphaFold-derived distance matrices for pathogens with low homology.

Attention-augmented CNNs [104] and saliency-based interpretation methods (e.g. Grad-CAM, integrated gradients [105]) can highlight interaction-relevant residues to address these issues. To address class imbalance, you can use weighted loss functions, focal loss, or biologically constrained sampling strategies to ensure fair representation of rare positives. The dual-branch or hierarchical CNN pipelines [77] encode host and pathogen proteins separately before the fusion process to avoid distortion caused by the differences in sequence length or motif density. Furthermore, adding structural inputs to sequence-based embeddings from huge protein language models stabilizes performance even in the presence of incorrect structural predictions.

### Recurrent neural networks

RNNs are a class of deep learning models specifically tailored for sequential data [106]. They are used in HP-PPI prediction due to their properties, which allow the identification of temporal and sequential dependencies [79]. Other advanced RNNs including LSTM networks and GRUs extend their capability to learn long-term dependencies and intricate interactions between the significant variables in contrast to basic RNNs [107]. An RNN processes protein sequences as input by maintaining recurrent connections that allow the model to remember previous inputs. The recurrence is expressed mathematically as


$$ {h}_t=f\Big({W}_h{h}_{t-1}+{W}_x{x}_t+{b}_h\Big) $$

${h}_t$ is the hidden state at time step $t$ encapsulating information up to $t$, and ${x}_t$ is the input at step $t$, typically an encoded amino acid representation. ${W}_h,{W}_x$ is the weight matrices for the hidden state and input, respectively, and $f$ is the activation function (e.g. ReLU). The final hidden state (${h}_T$) serves as the representation of the protein sequence, which is passed to the output layer for prediction. For binary classification tasks, the output layer computes the probability of interaction using


$$ \hat{y}=\sigma \left({W}_h.{h}_T+{b}_y\right) $$

Standard RNNs are affected by vanishing gradients, which makes it difficult for them to learn long-term dependencies. More advanced models, such as LSTMs and GRUs, mitigate these problems by using gates. These gating mechanisms enable LSTMs to incorporate long-range dependencies, which are essential for capturing relations, interactions, and sequences in protein sequences. Recently, Ahmed *et al*. [108] introduced a tree recurrent neural network with structured attention for PPI prediction, achieving state-of-the-art results without manual feature extraction. Yang *et al*. [70] also highlighted the role of RNNs in capturing long-term dependencies in protein sequences, complementing CNN-based approaches. Mewara *et al*. [109] proposed an RNN architecture with auto-feature engineering and layer-wise abstraction, showing improved performance on both intra- and inter-species datasets. Bonferroni *post hoc* analysis confirmed the statistical significance of their results [110]. While RNNs effectively model sequence data, their main limitation is poor interpretability [111]. Incorporating attention mechanisms or gradient-based XAI techniques may help clarify model decisions and support biological validation.

Another major limitation is that when RNNs are applied to very long host proteins, the resulting gradients either vanish or become unstable to the gating mechanisms. The hidden states and gating operations of RNN architectures do not reveal which residues or motifs drive a predicted interaction, resulting in poor interpretability. RNNs are not very computationally efficient when scaled to large host and pathogen proteomes. Standard RNNs are also unable to model cross-protein interactions directly as they encode sequences individually and subsequently fuse them using a secondary mechanism that may fail to capture co-evolving/complementary motifs.

By employing attention modules based on transformer architectures [112], the drawbacks may be sidestepped. These attention mechanisms could enable the model to learn long-distance relationships as an added benefit. Understanding a model better is possible through the use of attention weights and perturbation analysis. The combination of CNN and RNN offers an effective solution. The CNN layers find short-range features. While RNN layers capture the global context of sequences. In addition, dual-encoder or Siamese RNN architectures jointly encode host and pathogen sequences, resulting in more biologically coherent and effective cross-kingdom interaction representations for prediction.

### Graph neural network

Graph neural networks (GNNs) have improved HP-PPI predictions by incorporating graph-based representations of protein interaction graphs [111]. In GNNs, proteins are the nodes while the interactions are the edges, which facilitate the representation of both local and global interactions [113]. GNNs perform feature propagation over the neighbouring nodes and can learn multiple levels of abstraction, which is useful in comprehending the molecular behaviours. A graph convolutional layer aggregates information from neighbouring nodes to update each node’s feature representation. The updated feature for node $i$ at layer $l$:


$${h}_i^{\left(l+1\right)}=\sigma \Big({W}^{(l)}\sum_{j\epsilon N(i)}\frac{1}{c_{ij}}{h}_j^{(l)} \Big)$$

represents the neighbours of node while$i$  $\sigma$ is the activation function, and $l$ is the learnable weight matrix at the layer. After multiple convolutional layers, a graph-level embedding is generated by pooling information across all nodes. The pooled embedding is passed through fully connected layers to predict the likelihood of interaction between host and pathogen proteins. Fout *et al*. [67] used a GCN to model protein structures as graphs, achieving an AUC-ROC of 0.89. Zitnik *et al*. [114] applied GNNs to uncover effector-host interactions in *Salmonella*, while Wang *et al*. [115] used them to predict plant-pathogen interactions, aiding crop resistance research.

A major limitation of GNN-based HP-PPI prediction is its reliance on quality graph representations. Many pathogen proteins, such as secreted effectors, intrinsically disordered proteins, and viral proteins with no structural templates, lack accurate residue-residue contact maps or reliable AlphaFold structures. The incomplete, noisy, or biologically inconsistent nature of the graphs employed to train GNNs hinders the model’s capacity to learn meaningful structural or topological interaction cues. Host proteins are usually longer with more domains. These characteristics further enhance biases leading to extremely uneven graph sizes and connectivity. This inconsistency hampers cross-kingdom modelling and decreases predictive robustness. Using multimodal GNN architectures that combine sequence embeddings from large pretrained models (e.g. ESM, ProtT5), predicted contact maps, and functional annotations can help to fill missing or faulty graphs. The cross-modality inputs allow the model to maintain the biological relationships when the structure graphs are imperfect.

#### Graph convolutional networks

The most commonly used GNN architecture in early HP-PPI models must be graph convolutional networks. GCNs assign identical weights to all neighbours in message passing, which is biologically unrealistic, as only a subset of residues or domains mediates host-pathogen binding [116]. This averaging process clouds detailed interface information and may hide important effector motifs, catalytic sites, or immune-related motifs. Also, as we go deeper into layers of GCN, the node features become more alike, making it hard to differentiate. These limitations can be alleviated by incorporating transformer-based sequence embeddings [117] or by limiting the receptive field to biologically relevant neighbourhoods. Still, with the complexity of HP-PPI prediction, GNNs with alternative architectures are necessary to alleviate the GCNs’ inherent shortcomings.

#### Graph attention networks

Graph attention networks (GANs) improve on GCNs by assigning trainable attention weights to each neighbour, enabling the model to distinguish biologically relevant edges. This capacity has been demonstrated across multiple biological graph-learning studies. For example, Vrahatis *et al*. [118] provided a comprehensive review illustrating how GAT architectures enhance feature discrimination in complex biological graphs, highlighting their suitability for tasks involving uneven node importance. In microbe-host association prediction, Long *et al*. [119] introduced GATMDA. This GAT-based inductive matrix completion model significantly improved the prediction of human microbe-disease associations, demonstrating the ability of attention mechanisms to capture cross-entity relationships, an ability directly transferable to host-pathogen protein interactions. Similarly, Liu *et al*. [90] proposed MGATMDA. This multi-component GAT framework effectively integrated heterogeneous biological features to strengthen disease association prediction, underscoring the advantage of attention-driven message passing in biologically diverse systems. More recently, Liu *et al*. [120] developed PHPGAT, a multimodal heterogeneous GATv2-based model for phage-host prediction, further confirming that attention-augmented GNNs outperform GCNs when modelling interactions across fundamentally different biological entities. These advancements collectively validate the relevance of GATs for HP-PPI prediction, where cross-kingdom interactions involve highly asymmetric and motif-specific residue contributions that benefit from the selective weighing offered by attention mechanisms.

#### Generative models for HP-PPI prediction

The development of generative AI creates new frameworks that model host-pathogen protein interactions beyond the classical discriminative prediction. The host and pathogen protein representations themselves are learned by generative models. These models can generate new embeddings or perturbations that are compatible with interactions that mimic pathogen evolution. Variational autoencoders (VAEs) and generative adversarial networks (GANs) have been utilized to produce sequence embeddings enriched by structural or evolutionary constrains to improve model separability for asymmetric host-pathogen features [121]. Protein models based on diffusion, such as RFdiffusion [29], can excel at generating interaction-dimensional protein models. This enables the *in silico* generation of pathogen effector variants and their possible host targets. Graph-based generative models further extend this ability by sampling plausible edges within pathogen-host bipartite networks. The MGATMDA framework has shown that graph attention-driven generative sampling can enhance predictions of microbe-disease associations [122] and that similar approaches have been adapted recently for cross-kingdom PPIs. Yao *et al*. [123] showed that contrastive along with generative training improves identification of virulence-factor-associated interactions on pathogen PPI networks using GraphSAGE-based GNN backbones. Generative self-supervised protein models have also appeared. Generative models add capabilities not available in classical PPI workflows. For example, they enable the simulation of pathogen evolution, the *de novo* generation of new interfaces, and the augmentation of scarce HP-PPIs. Additionally, generative models are very relevant for studying emerging pathogens.

Furthermore, generative models play an emerging, increasingly important role in HP-PPI prediction by learning latent representations that capture hidden structural, evolutionary, or functional relationships between interacting proteins, particularly when data are sparse or incomplete. Models that are trained to learn the distribution of host and pathogen sequence features rather than just interaction labels can predict novel interactions that are plausible. The power of this approach is demonstrated in recent work in viral immunology. For instance, Bist and associates employed a GAN to model immune-evasive SARS-CoV-2 spike mutations. So, they produced synthetic spike sequences with escape properties of natural variants [124]. Using these GAN-generated sequences in a downstream prediction model improved escape-mutation detection on the Greaney dataset by 7%. This typically uncovered hidden evolutionary routes and improved prediction in low-resource viral settings. Also, Wang *et al*. [124] designed a deep molecular generative framework for PPI-targeting inhibitors. Generative latent embeddings can capture biochemical features that define the PPI inhibitor space and generate novel drug-like compounds. Even though the study relates to PPI inhibition at the ‘therapeutic’ level rather than the ‘host-pathogen’ level, it displays that generative latent modelling can generate extrapolations from the known to the unknown, which can relate to innovation in solution space that is complementary to what is already in the current PPI situation. A combination of the examples presented above demonstrates that generative models can infer latent interaction relationships, complete missing edges in incomplete HP-PPI networks, and generate new therapeutic hypotheses, which may be particularly advantageous for pathogens evolving rapidly or in systems with few experimentally validated interactions.

#### GraphSAGE (inductive neighbourhood sampling GNNs)

GraphSAGE goes beyond the capabilities of the GCN architecture. The architecture is designed to sample nodes inductively from the neighbourhood of the already seen nodes. This is essential in HP-PPI prediction as they do not always have complete structures or interactions. Multiple recent studies support its use in modelling cross-kingdom interactions. Koca *et al*. [116] find that GraphSAGE, which leverages sampled neighbourhoods, outperformed standard GCNs in predicting virus-human PPIs for novel viral species, even when sufficient evidence of interaction was absent. A later paper by the same authors [125] applied GraphSAGE to PHI (Pathogen-Host Interaction) networks, which again outperformed classical convolution-based models in discriminative performance due to its ability to take advantage of local topological patterns without requiring the whole graph. Yao *et al*. [123] used GraphSAGE as a backbone in a framework for generating and contrasting virulence factors. This makes them robust across bacterial datasets. This also shows that GraphSAGE can learn informative representations when there is not enough data. All of the studies show that GraphSAGE inductive sampling and flexible aggregation strategies are beneficial for HP-PPI prediction. The protein graph landscape is sparse, incomplete, and evolving across pathogens.

#### Models with multiple relationships are HetGNN and R-GCN

The interactions between a pathogen and a host involve several biological relationship types, including functional similarity, evolutionary proximity, domain co-occurrence, and predicted contact edges. Traditional GNNs use edges that are the same and cannot put in heterogeneous biological evidence. Models from heterogeneous GNNs, such as relational GCNs (R-GCN) [126] and HetGNN [127], support for multi-type nodes and edges. We can thus explicitly model the sequence-level, structural, functional, and pathway relationships. These architectures are highly advantageous for predicting HP-PPIs, and host and pathogen proteins differ fundamentally in their evolutionary background and molecular properties. On the other hand, heterogeneous GNNs require tailored relational schemas and consume substantial memory. If relational definitions are incomplete or contradictory, the model may pass on false information. Some of the solutions could be automating relation extraction from any available domain database (Pfam, InterPro) or using sparsified edge sets or using pretrained embeddings to lessen the reliance on dense multi-relational graphs.

#### Pooling-based GNNs (DiffPool, TopK, SAGPool)

Hierarchical GNNs use pooling techniques to condense residue-level graphs into domain- or protein-level representations, mirroring the structure of actual biological systems. In HP-PPI modelling, it is influenced by modular domains rather than single elongated residues. Methods such as DiffPool [35], TopK pooling [36], or SAGPool [37] help the network learn coarse-grained structures. This allows the network to capture biological meaningful patterns like effector domains, leucine-rich repeats, or kinase lobes. However, hierarchical pooling can increase computation and sometimes generate biologically ambiguous clusters, they do not match real structural modules. Also, without appropriate regularization, pooling layers may remove essential residues from interfaces but retain other. Using hierarchical GNNs with attention, adding structural priors (e.g. binding-site annotations), and constructing multi-resolution graphs can alleviate these problems. Using hierarchical GNNs can give a powerful, multi-scale perspective that simpler architectures do not offer.

## Hybrid and ensemble models

The use of hybrid models allows different feature extraction techniques or algorithms to be combined to improve prediction accuracy and interpretability [128]. They start with feature extraction from sequence data (e.g. k-mers, PSSM), structural data (e.g. contact maps), or functional data (e.g. GO, pathways) [129]. These features are then fused together through concatenation, weighted averaging, or attention mechanisms before being passed onto models. There are several distinct architectures within hybrids: in layered models, feature extraction is performed with CNNs, and prediction is performed with Random Forests, while in parallel models, features are computed in parallel before their outputs are merged. In Ensemble models, on the other hand, the individual predictions from multiple learners are combined to increase robustness. These include bagging (e.g. Random Forest [96]), boosting (e.g. Gradient Boosted Trees [130]), and stacking, where one meta-learner is used to merge multiple model outputs. Recent studies underscore their importance: HPiP, which relied on an ensemble of SVM, RF, and LR integrative classifiers, accurately predicted SARS-CoV-2 host interactions, validated by AP/MS experiments [131]. Mahapatra and Sahu also built a hybrid model that fused the FSNN and the LGBM framework using pseudo amino acid and conjoint triad features and showed consistently high accuracy across all datasets. As a whole, the two approaches, notably hybrid and ensemble models, manage the complexity relating to host-pathogen PPI prediction more reliably for aiding the understanding of diseases and discovering new therapies.

## Advanced strategies for improving HP-PPI prediction

Recent studies have shown that classical machine learning and simple underlying deep architectures are not enough to spot host-pathogen interactions. As a result, newer HP-PPI models rely more on transfer learning, explainable AI (XAI), and multi-omics data integration, thereby enhancing generalization, mechanistic interpretability, and biological context.

### Transfer learning for cross-kingdom generalization

Transfer learning has become a powerful technique for predicting HP-PPIs, especially when there are not enough experimentally confirmed interactions. Protein language models like ESM-2 [78], ProtTrans [132], and MSA Transformer [133] learn structural and evolutionary constraints from millions of proteins, thus allowing rich cross-kingdom feature representation. Using these pretrained embeddings greatly enhances performance for models making predictions of interactions with new viruses, emerging fungal pathogens, or neglected bacterial species. Multiple studies that subscribe to transfer learning have improved downstream performance in viral-host PPI prediction [134], pathogen effector classification (Effector-GAN) [135], and receptor-ligand interaction modelling [136], which supports robust HP-PPI inference. Using pretrained embeddings in CNN, RNN, or GNN model architectures helps in the recognition of conserved signatures of the interface even when there is low host-pathogen protein sequence similarity.

### Explainable AI for biological interpretability

Interpretable predictions of HP-PPI models must be mechanistically validated in the wet laboratory. To work on this, modern HP-PPIs increasingly use XAI, e.g. SHAP values, integrated gradients, Grad-CAM, GNNExplainer, etc. Residue-level contribution scores are provided by SHAP [137]. These scores help to highlight host receptor patches or pathogen effector motifs responsible for the interaction signal. Grad-CAM [105] was used to see which regions were discriminative in a sequence-based CNN, while GNNExplainer [138] was used to find subgraph structures corresponding to binding interfaces in a protein contact network. HP-PPI models can produce interpretable hypotheses, e.g. virulence-associated loops in bacterial effectors or kinase-binding submotifs in host proteins. Together, these approaches link high-throughput computational predictions with experimental design.

### Multi-omics integration for context-specific HP-PPI discovery

The interaction between the host and the pathogen occurs in a dynamic environment. This environment is influenced by the pathogen’s gene expression, protein abundance, post-translational modifications, and cellular localization. Integrating multi-omics modalities like RNA-seq, proteomic, phosphoproteomic, epigenomic, and metabolomic greatly enhances HP-PPI prediction context. Combining different ‘omics’ has improved disease modelling for infectious diseases [139], accurately predicted effector-target interactions in plant pathogens, and enabled immune pathway reconstruction [140, 141]. Multimodal GNNs, hierarchical late-fusion neural networks, as well as attention-based cross-omics integrators are models that allow HP-PPI frameworks to incorporate an infection-stage specificity, e.g. capturing early innate immune interactions that differ from late-stage intracellular ones. Combining various omics data helps to remove misleading results. It also makes the analysis more biologically relevant and usable. These developments (Table 2) enhance HP-PPI prediction by introducing more data-efficient, interpretable, and biologically contextualized models to the field of sequence-based learning.

**Table 2:** Integrating transfer learning, explainable AI, and multi-omics for next-generation HP-PPI prediction

Approach	Significant	Relevance to HP-PPI prediction	Key tools/methods	Limitations	Proposed solutions
**Transfer learning**	Uses pretrained protein language models to provide rich evolutionary and structural representations learned from millions of protein sequences	Improves cross-kingdom generalization; enables accurate HP-PPI prediction for novel or poorly characterized pathogens; reduces need for large labelled datasets	ESM-2, ProtT5, MSA Transformer; pretrained embeddings integrated into CNN/RNN/GNN architectures	Limited availability of pathogen-specific embeddings; transfer bias when host-pathogen divergence is high	Domain-adaptation training, contrastive learning, pathogen-aware fine-tuning, hybrid sequence + structure embeddings
**Explainable AI (XAI)**	Methods that provide human-interpretable explanations of model decisions, highlighting residues, domains, or structural regions important for prediction	Enhances biological interpretability; helps identify effector motifs, host receptor patches, virulence residues; supports downstream experimental validation	SHAP, Integrated Gradients, Grad-CAM, GNNExplainer, PGExplainer	Some XAI outputs are coarse or noisy; interpretation varies across models; difficult to map explanations to wet-laboratory mechanisms	Use ensemble XAI, residue-level saliency filtering, and structure-aware XAI; integrate AlphaFold models to anchor explanations
**Multi-omics integration**	Combines multiple biological layers, transcriptomics, proteomics, phosphoproteomics, metabolomics, epigenomics, to contextualize interactions during infection	Captures infection-stage dynamics; improves biological realism; identifies condition-specific HP-PPIs (e.g. early innate versus late adaptive interactions)	Multimodal deep learning, graph-based integration, hierarchical late-fusion, attention-based cross-omics networks	Omics datasets vary in scale and noise; integration can introduce redundancy; lacks standardized preprocessing pipelines	Feature-level normalization, graph fusion strategies, domain-specific weighting, stage-specific learning and time-series omics

### Pretrained protein language models in HP-PPI prediction

Protein language models (pLMs) represent protein sequences. These models capture the evolution, structure, and function features from sequence corpora. ESM-2, ProtT5, and MSA transformer are a few of these models that were pretrained. With respect to predicting host-pathogen interactions, these models provide a viable avenue to address two critical issues: (1) the marked sequence divergence that exists between hosts and pathogens, and (2) the lack of experimental data on cross-kingdom pairs. Recent studies confirm their utility as foor instance, Jiang *et al*. [142] propose a framework for human-virus PPI prediction by linking pLMs with graph-based structural features, yielding improved AUC compared to traditional embeddings. According to Liu *et al*., a model was developed that uses pLM embeddings and multiple instance learning to predict virus-host interactions. Using pLM-based features shows higher recall and generalization to novel viruses [143]. While direct large-scale HP-PPI tools based on pLMs are still evolving, our findings suggest that embedding host and pathogen proteins via pLMs and then applying classifiers (or GNNs) improves performance in cross-kingdom settings. Nevertheless, several gaps remain. A lot of pLM-based models are trained on intra-species PPIs, or model systems where plenty of data are available, rather than on truly novel pathogens, or leave-one-pathogen-out validations. Also, while pLMs are good at representation, effective architectures for modelling interaction pairs (versus single proteins) are still under development (e.g. pair-input fine-tuning). Bringing together pLM embeddings combined with domain-specific fine-tuning, cross-kingdom contrastive learning, and incorporation into GNNs or hybrid architectures can help the future HP-PPI predictors. To conclude, pretrained protein language models emerged as a powerful and increasingly used tool in HP-PPI prediction, making their inclusion in this review useful and actionable for next-generation modelling.

## Benchmarking performance and practical applicability of HP-PPI predictors

### Quantitative performance of representative HP-PPI predictors

A major limitation in the current HP-PPI literature is the lack of a unified benchmark that compares prediction methods across consistent datasets and evaluation protocols. To address this gap, we incorporated a quantitative summary of published performance results reported for representative HP-PPI models. The deepHPI platform, developed by Kaundal *et al*. [144], is a CNN-based tool that has been built on HPIDB-derived interactions for different categories, which include plant-pathogen, human-bacteria, human-virus, and animal-pathogen. Upon testing on validation sets, deepHPI achieved performances of 98.91% for plant-pathogen, 95.73% for human-bacteria, 99.29% for human-virus, and 97.49% for animal-pathogen. The Matthews correlation coefficients (MCC) were 0.94 (plant-pathogen), 0.75 (human-bacteria), 0.96 (human-virus), and 0.87 (animal-pathogen), indicating that well-tuned CNNs can reach very high discriminative power. In addition, Deep-HPI-pred [145] showed performance on four host-pathogen systems using graph-derived features and ensemble learning on the R-Shiny platform for network-based HP-PPI prediction. The AUCs reported on curated benchmarks were accuracies of 98.4%, 97.9%, 94.3%, and 96.6% for plant-pathogen, human-bacteria, human-virus, and animal-bacteria datasets, respectively, with MCC values more than 0.80 in all cases. From these findings, we conclude that network topology, along with classical sequence- and function-based descriptors, works best for HP-PPI classification.

Yang and colleagues [146] put forward a model. This model created a doc2vec + Random Forest. It embeds sequences of viral and human proteins. A tree-based classifier then uses the embedding. This model achieves an AUC of 0.947 on a widely used human-virus benchmark using five-fold CV, outperforming several previous feature-engineering pipelines. Tsukiyama *et al*. [147] proposed LSTM-PHV. This model is designed to learn contextual sequence patterns. It uses word2vec embedding and bidirectional LSTMs. The LSTM-PHV model achieved an AUC of 0.976 and an accuracy of 98.4% in five-fold cross-validation. In addition, on an independent human-virus test set, this model outperformed several existing predictors, DeepViral, doc2vec-based predictors, and others.

In another relevant work, Dong *et al*. [148] proposed a multitask transfer-learning framework for virus-human PPIs. In their model, a shared representation is jointly trained across different viral species and then specialized for each virus and test their approach later on human-virus benchmarks. On these benchmarks, author reported that their method consistently improves AUC and average precision (AP). This is compared to single-task baselines, including doc2vec + RF and DeepViral and works particularly well in low-data settings. It demonstrates the concrete benefit of transfer learning in the case of under-sampled pathogens.

All these findings suggest that the state-of-the-art HP-PPI models typically produce AUC scores in the range of 0.94 to 0.99 and MCC scores of ≥0.75, which is definitely a good score on functional, curated benchmarks involving human, animal, and plant systems. Still, a large share of reported metrics is obtained under relatively controlled conditions (fixed host species, restricted viral or bacterial families, well-curated negatives).

### Real-world applicability of HP-PPI predictors

Research findings from recent biological applications show that HP-PPI models are computationally accurate and practically useful. At the onset of the COVID-19 pandemic, computational analyses were conducted to prioritize human proteins that interact with SARS-CoV-2 viral factors. Several predicted interactions, including NSP13 with TBK1 and NSP1 with eIF3, were subsequently experimentally validated using affinity purification, mass spectrometry, and CRISPR knockout screening [69, 149]. Studies quickly identified pathways with host dependence and added to the early repurposing of antiviral drugs. Utility has also been demonstrated in plant and pathogen systems. A study of bacterial infection biology HP-PPI predictions has yielded mechanistic insight into host signalling manipulation. For example, computational predictions of Salmonella effector SopB interacting with host proteins were later experimentally validated. Garcia-Gill’s study demonstrates that the *Salmonella* effector SopB activates the PI3K-PDK1-mTORC2-Akt-YAP signalling pathway in B cells to suppress NLRC4 inflammasome activation, thereby creating a survival niche that promotes bacterial persistence [150]. Although HP-PPI predictors have achieved useful results, they are not yet considered substitutes for experimental interactome mapping. They are best viewed as useful tools for prioritization and hypothesis generation. Two major limitations remain unresolved. First, negative samples in HP-PPI datasets are usually assumed rather than experimentally validated, creating uncertainty that can artificially inflate machine learning performance [144]. Second, interactions between the pathogen and the host are not very strongly evolutionarily conserved either. This is because many pathogenic effectors evolve rapidly and independently from the host. The lack of evolutionary conservation weakens sequence-based prediction models. An example of such a prediction model is the protein language model. So the best approach for now is a hybrid workflow where computational models identify candidates with high-confidence structural predictors (e.g. AlphaFold-Multimer, AlphaFold3), structural plausibility is evaluated, and experimental methods (yeast two-hybrid, co-immunoprecipitation, AP-MS, etc.) provide final confirmation. This combined approach has been more successfully applied to viral, bacterial, and plant-pathogen systems and today represents the most realistic translational use case for HP-PPI predictors.

## Challenges and advanced approaches for resolution

There are many difficulties with predicting biological HPIs. The problems stem from both biological complexity and computational limits. One major problem is the lack of data. There are very few validated HP-PPI datasets because high-throughput validation is expensive, tedious, and usually specific to a particular pathogen [151]. This problem is particularly severe when dealing with understudied pathogens. Class imbalance is another significant problem, as the non-interacting pairs heavily outnumber the interacting pairs (Supplementary File 1: Table S3). Often, the negative samples are the only ones considered, which can lead to false negatives. Differences between species also complicate cross-organism transfer of knowledge regarding protein interactions. The presence of organism-specific immune-evasion mechanisms also adds another level of difficulty to predictions. The combination of multiple data types, such as structural, functional, and sequence data, also increases the computational complexity of processing this data. Recent advancements in structural biology, particularly AlphaFold-Multimer, have demonstrated robust performance in the modelling of protein–protein complexes, including cross-kingdom host-pathogen pairs. AlphaFold3 now implements improved diffusion-based modelling and multi-chain structural refinement, enabling it to model interface geometry with greater confidence. Abramson *et al*. [152] reported benchmarks for multimers showing an accuracy improvement of 20%-30% over AlphaFold-Multimer’s predictions. In addition, this prediction can certainly recover several viral-host interaction complexes with quality close to that of experiments. AlphaFold3 cannot replace machine-learning HP-PPI predictors, but it does have limitations. In the first place, AlphaFold3 cannot independently determine whether two proteins interact, as it still requires a candidate protein pair as input. Unlike structural models, HP-PPI prediction methods evaluate millions of candidate host-pathogen interactions, a scale that current models cannot support. Moreover, AlphaFold3 performance slows down when proteins that interact with each other do not exhibit strong coevolutionary signals. This holds great promise for host-pathogen systems where the pathogen effector evolves rapidly, horizontally, and independently of host proteins. Many viral or bacterial effectors have not evolved sufficiently over time to be accurately modelled. Third, it is computationally intensive and therefore not suitable for interactome screening at the genome scale, where sequence-based deep learning and graph-learning models are much more efficient. Besides, structural prediction does not directly address other HP-PPI complexities, such as tissue specificity, infection-stage regulation, multi-omics constraints, and subcellular localization, which machine-learning predictors better capture. Because of this, AlphaFold3 is a good validation tool, but not a substitute. A pipeline integration may represent a realistic future direction, with ML-based HP-PPI predictions used for candidate prioritization (by narrowing down candidates), followed by AlphaFold3 for structural refinement and experimental planning. The hybrid approach has already delivered encouraging results in small case studies concerning viral-host interactomes.

Recent developments such as transfer learning, deep learning, and improved negative sampling are more likely to solve the problem. For instance, transfer learning helps address data scarcity by enabling models trained on well-studied systems to be applied to new pathogens, thereby improving generalizability. In a similar study, Yang *et al*. [70] proposed a multi-scale convolutional neural network framework that utilized transfer learning to predict human-virus PPIs. The model fine-tuned parameters from general PPI datasets to specific viral datasets with the aim of gaining significant improvements in accuracy, which showed that transfer learning could be useful for dealing with data availability. Durmus *et al*. [153] exploit extensive knowledge of protein sequences and human interactome patterns to predict interactions related to novel pathogens. Another approach to handling HP-PPI dataset heterogeneity is to use deep learning methods, such as CNNs and RNNs, which automate the extraction of complex features from protein sequences and structures. Such methods can integrate diverse data modalities, such as sequence embeddings, structural features, and functional annotations, into a unified predictive framework. For instance, CNNs excel at identifying spatial and sequential patterns in protein sequences, while RNNs effectively capture long-range dependencies. Hybrid architectures that combine both approaches further improve model performance by leveraging the strengths of each method. The importance of precision in adverse sample selection directly correlates with reliable HP-PPI prediction, as mislabelled non-interacting pairs introduce noise. With the sophisticated methods now available, it is possible to filter out protein pairs that show any indication of interaction, thereby increasing model precision and recall, as demonstrated by Kaundal *et al*. [144]. Use of XAI alongside GNN enhances interpretability, extending the reach of AI models of host-pathogen interactions, which eases the process of target recognition. Regardless of the problems encountered, the pace of development in deep and transfer learning, as well as in data collection and organization, continues to increase prediction accuracy and biological relevance, and to enable the development of therapies of higher precision.

## Conclusion

The prediction of host-pathogen protein-protein interactions is critical for understanding infection mechanisms and developing a treatment plan. It comes with limitations, such as insufficient data, class imbalance, and a model that lacks sufficient depth. Recent technologies such as transfer learning, hybrid and ensemble models, and XAI addressed these issues, by improving the accuracy and interpretability of PPI predictions. Transfer learning leverages knowledge from well-studied systems and applies it to understudied pathogens, while hybrid models such as CNNs and GNNs help analyze a variety of data. XAI enables the prediction of outcomes from biological processes by applying a transparent approach to the model’s decision-making. Now, techniques for data integration combine sequence, structure, and functional cognition to integrate more raw data with concrete executable information. Predefined, standardized benchmarks alongside curated datasets increase robustness, and attention mechanisms combined with ensemble methods help minimize complexity and reduce false-positive outcomes. With the rise of novel pathogens, ease of adaptation alongside clarity in the models will be invaluable features. Advancing the speed at which diagnostics, vaccines, and treatments are developed will rely on integrating multi-omics, real-time learning, and expanding open-access datasets.

**Key Points**

- Transfer learning significantly improves host-pathogen protein-protein interaction (HP-PPI) prediction for less-characterized pathogens by leveraging insights from well-studied host-pathogen systems.
- Hybrid and ensemble machine learning models enhance predictive accuracy by integrating the strengths of multiple algorithms.
- Explainable AI (XAI) provides interpretability to computational predictions, offering biologically meaningful insights into protein-protein interactions.
- Multi-omics data integration and graph-based learning approaches enrich model inputs, leading to more robust and biologically relevant predictions.
- The field is moving towards scalable, interpretable, and data-driven frameworks that support therapeutic discovery, vaccine development, and precision medicine in infectious disease research.

## Supplementary Material

Supplementary_File_1_bbag051

## Data Availability

The data underlying this article are available in the article and in its online supplementary material.
