# Supplementary material for: Comprehensive review and assessment of machine learning approaches for host-pathogen protein-protein interaction prediction
Source: Brief Bioinform. 2026 Feb 10;27(1):bbag051. doi: 10.1093/bib/bbag051 (PMC12888821; doi:10.1093/bib/bbag051)
Supplement: Supplementary_File_1_bbag051 [file supplementary_file_1_bbag051.docx]

**Table S1:** Experimental techniques for Protein-Protein Interactions (PPIs)

| **Technique** | **Key Benefits** | **Challenges** | **References** |
| --- | --- | --- | --- |
| **Yeast Two-Hybrid (Y2H)** | - High throughput for large-scale screening. - Can identify direct binary interactions. | - High false-positive rates, requiring validation. - Limited detection of transient or weak interactions. - Inadequate for post-translational modifications in host proteins. - Poor performance for membrane proteins. | [1] |
| **Mammalian Two-Hybrid (M2H)** | - Detects PPIs under near-physiological conditions. - Accommodates mammalian-specific modifications. | - Requires optimized mammalian cell systems. - Limited scalability compared to Y2H. - Transient interactions remain challenging. | [2] |
| **Co-Immunoprecipitation (Co-IP)** | - Suitable for studying PPIs in their native cellular context. - High specificity with good antibodies. | - Labor-intensive and low throughput. - Requires high-quality antibodies. - Poor detection of transient interactions. - Risk of losing weak interactions during washing steps. | [3] |
| **Affinity Purification-Mass Spectrometry (AP-MS)** | - High specificity and sensitivity for protein complexes. - Can identify multiple interaction partners simultaneously. - Provides quantitative data. | - Requires significant expertise and resources. - Loss of transient or weak interactions during purification. - Complex data interpretation. - Potential for non-specific binding with cross-linking. | [4] |
| **Chemical Cross-Linking (with MS)** | - Stabilizes transient or weak interactions. - Provides structural insights into interaction interfaces. | - Can introduce non-specific cross-links. - Data interpretation is computationally intensive. - Requires careful optimization of cross-linker. | [5] |
| **Proximity Labeling (e.g., BioID, APEX)** | - Detects transient and weak interactions. - Captures PPIs in live cells and native environments. - Suitable for membrane proteins. | - Potential labeling of non-specific nearby proteins. - Requires optimization of labeling conditions. - Dependency on fusion constructs and controls. | [6] |
| **Fluorescence Resonance Energy Transfer (FRET)** | - Allows real-time monitoring of PPIs in live cells. - High spatial resolution for dynamic studies. | - Requires precise protein labeling. - Limited to interactions within a specific proximity range. - Challenging for large-scale studies. | [7] |
| **Bimolecular Fluorescence Complementation (BiFC)** | - Visualizes PPIs in specific subcellular locations. - Suitable for live-cell imaging. | - Irreversible reconstitution may cause artifacts. - Limited for transient interactions. - Requires large-scale imaging for high-throughput studies. | [8] |
| **Surface Plasmon Resonance (SPR)** | - Provides real-time kinetic data (binding affinities, association, and dissociation rates). | - Requires purified proteins. - Limited to direct binary interactions. - High cost for instrumentation. | [9] |
| **Protein Microarrays** | - High-throughput screening for thousands of interactions. - Versatile for testing various interaction types. | - Protein immobilization may affect function. - High cost and technical complexity. - Limited to stable interactions. | [10] |
| **X-Ray Crystallography** | - Gold standard for structural analysis. - Reveals atomic details of interaction interfaces. | - Requires crystallization of protein complexes. - Not suitable for dynamic or transient interactions. - Time-consuming and resource-intensive. | [11] |
| **Cryo-Electron Microscopy (Cryo-EM)** | - Provides structural data for large complexes. - Suitable for heterogeneous samples. - No need for crystallization. | - Requires advanced instrumentation and expertise. - Limited resolution compared to crystallography for small proteins. - Expensive setup and maintenance. | [12] |
| **Single-Molecule Techniques (e.g., smFRET)** | - High sensitivity to transient interactions. - Enables dynamic analysis of interaction processes. | - Requires specialized equipment and expertise. - Limited throughput for large-scale studies. - Fluorescence labeling can alter protein behavior. | [13] |

**Table S2:** Feature selection in host-pathogen PPI studies

| **Feature Selection Method** | **Description** | **Applications in Host-Pathogen PPIs** | **Benefits** | **Challenges** |
| --- | --- | --- | --- | --- |
| **Filter Methods** | Evaluates each feature independently using statistical metrics (e.g., correlation, mutual information). | - Identifying sequence motifs (e.g., k-mers or conserved amino acids) relevant to host-pathogen binding. - Ranking physicochemical properties for distinguishing interacting and non-interacting proteins. | - Computationally efficient. - Works well with high-dimensional data like sequences. | - Ignores feature interactions. - May miss context-specific features important for cross-species interactions. |
| **Wrapper Methods** | Uses the ML model to evaluate subsets of features iteratively, optimizing for predictive performance. | - Selecting structural features (e.g., docking scores or interaction hotspots) in host-pathogen systems. - Evaluating combinations of sequence and functional features for bacterial effector-host interactions. | - Captures feature dependencies. - Optimized for specific host-pathogen systems. | - Computationally intensive for large datasets. - Risk of overfitting when datasets are small. |
| **Embedded Methods** | Integrates feature selection into model training (e.g., decision trees or L1 regularization). | - Identifying important residues or functional annotations (e.g., Gene Ontology terms) during training. - Selecting key structural and sequence features in viral-host interactions (e.g., SARS-CoV-2 studies). | - Efficient since it combines selection and training. - Often yields high predictive accuracy. | - Requires high-quality datasets. - Computationally expensive for large-scale host-pathogen datasets. |
| **Mutual Information** | Measures dependency between features and the target variable (e.g., interaction likelihood). | - Capturing sequence and evolutionary features that indicate interaction interfaces in bacterial-host systems. - Detecting functional overlaps in viral proteins and host immune regulators. | - Captures non-linear relationships. - Useful for sequence-based studies. | - Computationally intensive for large datasets. - May select redundant features. |
| **Principal Component Analysis (PCA)** | Reduces dimensionality by transforming correlated features into uncorrelated components. | - Reducing the complexity of large datasets (e.g., viral interactomes). - Integrating diverse datasets, such as multi-omics data, to focus on the most informative components. | - Reduces noise while retaining significant variance. - Enables visualization of high-dimensional data. | - Difficult to interpret components in biological terms. - May overlook important non-linear patterns. |
| **Gene Ontology (GO) Enrichment Analysis** | Selects features based on GO term enrichment in interacting proteins. | - Prioritizing host-pathogen PPIs involved in immune pathways (e.g., inflammation or cytokine signaling). - Identifying functional overlaps in bacterial effectors targeting host cell processes. | - Provides biological interpretability. - Highlights pathway-specific interactions. | - Limited by the completeness of GO annotations. - Functional annotations may be noisy or inconsistent. |
| **Boruta Algorithm** | Compares the importance of features with randomized noise features to identify relevant ones. | - Robustly selecting cross-species interaction features, such as docking scores or functional similarity metrics. - Identifying key sequence patterns in viral proteins targeting host factors. | - Effective in identifying relevant features. - Robust to overfitting. | - Computationally expensive. - Time-consuming for high-dimensional host-pathogen datasets. |
| **Correlation-Based Feature Selection (CFS)** | Evaluates subsets of features based on their correlation with the target variable and their inter-correlation. | - Selecting non-redundant features in bacterial-host interaction datasets. - Reducing multicollinearity in physicochemical and functional features for viral-host interactions. | - Reduces redundancy in selected features. - Improves interpretability of feature sets. | - May exclude useful features that have low individual correlation but high combined relevance. |
| **t-Statistic / ANOVA** | Measures the difference in feature distributions between interacting and non-interacting proteins. | - Identifying distinguishing sequence features in host-pathogen PPIs (e.g., enriched residues in binding interfaces). - Selecting important structural features in transient interactions. | - Simple and interpretable. - Effective for identifying discriminatory features. | - Assumes normal distribution of features. - May miss non-linear dependencies in feature interactions. |
| **Domain-Based Selection** | Selects features based on known domains or motifs associated with interactions. | - Highlighting SH2 domains in host immune proteins targeted by viral effectors. - Prioritizing bacterial effectors with specific secretion system motifs targeting host cell machinery. | - Biologically meaningful. - Reduces noise by focusing on known interaction drivers. | - Requires prior knowledge of domain relevance. - May miss novel interaction domains. |

**Table S3:** Key challenges in HP-PPI prediction and presents advanced computational and experimental approaches for overcoming these obstacles.

| **Challenges** | **Description** | **Advanced approaches for resolution** |
| --- | --- | --- |
| **Data scarcity** | Limited availability of experimentally validated HP-PPI datasets. | Transfer learning for pretrained models on large human PPI datasets can be fine-tuned for specific pathogen-host systems. |
| **Class imbalance** | Non-interacting protein pairs vastly outnumber interacting pairs in datasets. | Weighted loss functions assign higher penalties to misclassifications of interacting pairs. Synthetic Data Generation: Use SMOTE to balance datasets. |
| **Negative sample selection** | Lack of reliable non-interacting protein pairs for training models. | Statistical sampling for selection of negatives with no known or indirect interactions. Curated Databases: Use high-confidence interaction data from resources like HPIDB. |
| **Cross-species variability** | Differences in protein structures and functions between species limit transferability of data. | Evolutionary features to incorporate conserved motifs and orthologous data to bridge gaps. Domain-specific models to train models for individual pathogens. |
| **Heterogeneous data types** | Diverse data types, such as sequences, structures, and functional annotations, require complex handling. | Hybrid models combine CNNs for sequence features and GNNs for network structures. Data fusion techniques use attention mechanisms to integrate diverse data types. |
| **Interpretability of models** | Deep learning models are often "black boxes" with limited biological interpretability. | Explainable AI (XAI) use attention mechanisms (e.g., Grad-CAM) and feature importance analysis to interpret predictions. |
| **Computational complexity** | Training models on large-scale interactomes is resource-intensive. | Use sparse matrix representations and sampling-based GNNs (e.g., GraphSAGE). Cloud and distributed computing utilize cloud resources to parallelize computations. |
| **High false positive rates** | Many predicted interactions may not be biologically relevant. | Use docking simulations or experimental assays to validate high-confidence predictions. |
| **Rapid pathogen evolution** | Pathogens evolve quickly, leading to altered interaction mechanisms. | Continuous learning models to regularly update models with newly available interaction data. Use ensemble models to account for variability. |
| **Lack of cross-validation standards** | Variability in validation methodologies leads to inconsistent benchmarks. | Develop standardized datasets and evaluation metrics for HP-PPI models. |

**References**

1. Mehla, J., J.H. Caufield, and P. Uetz, *The yeast two-hybrid system: a tool for mapping protein–protein interactions.* Cold Spring Harbor Protocols, 2015. 2015(5): p. pdb. top083345.

2. Lievens, S., et al., *The use of mammalian two-hybrid technologies for high-throughput drug screening.* Methods, 2012. 58(4): p. 335-342.

3. Ryu, J.Y., et al., *Profiling protein–protein interactions of single cancer cells with in situ lysis and co-immunoprecipitation.* Lab on a Chip, 2019. 19(11): p. 1922-1928.

4. Chen, G.I. and A.-C. Gingras, *Affinity-purification mass spectrometry (AP-MS) of serine/threonine phosphatases.* Methods, 2007. 42(3): p. 298-305.

5. Jiao, F., et al., *Exploring an alternative cysteine-reactive chemistry to enable proteome-wide PPI analysis by cross-linking mass spectrometry.* Analytical chemistry, 2023. 95(4): p. 2532-2539.

6. Béganton, B., et al., *Protein interactions study through proximity-labeling.* Expert Review of Proteomics, 2019. 16(8): p. 717-726.

7. Fernández-Dueñas, V., et al., *Fluorescence resonance energy transfer-based technologies in the study of protein–protein interactions at the cell surface.* Methods, 2012. 57(4): p. 467-472.

8. Miller, K.E., et al., *Bimolecular fluorescence complementation (BiFC) analysis: advances and recent applications for genome-wide interaction studies.* Journal of molecular biology, 2015. 427(11): p. 2039-2055.

9. Abouhajar, F.B., *Advanced Surface Plasmon Resonance (SPR) Techniques for Peptide-Based Inhibition Study and Multi-Parametric Cell Analysis*. 2022, University of California, Riverside.

10. Casado-Vela, J., M. Fuentes, and J.M. Franco-Zorrilla, *Screening of protein–protein and protein–DNA interactions using microarrays: Applications in biomedicine.* Advances in protein chemistry and structural biology, 2014. 95: p. 231-281.

11. Sijbesma, E., et al., *Structure-based evolution of a promiscuous inhibitor to a selective stabilizer of protein–protein interactions.* Nature communications, 2020. 11(1): p. 3954.

12. Chowdhury, S., et al., *Structural proteomics, electron cryo-microscopy and structural modeling approaches in bacteria–human protein interactions.* Medical microbiology and immunology, 2020. 209(3): p. 265-275.

13. Byron, O. and B. Vestergaard, *Protein–protein interactions: A supra-structural phenomenon demanding trans-disciplinary biophysical approaches.* Current opinion in structural biology, 2015. 35: p. 76-86.
